# Genoprotective activity of the *Pleurotus eryngii* mushrooms following their *in vitro* and *in vivo* fermentation by fecal microbiota

**DOI:** 10.3389/fnut.2022.988517

**Published:** 2022-08-23

**Authors:** Athina Boulaka, Panagiota Mantellou, Gabriela-Monica Stanc, Efthymia Souka, Christoς Valavanis, Georgia Saxami, Evdokia Mitsou, Georgios Koutrotsios, Georgios I. Zervakis, Adamantini Kyriacou, Vasiliki Pletsa, Panagiotis Georgiadis

**Affiliations:** ^1^Laboratory of Environment and Health, Institute of Chemical Biology, National Hellenic Research Foundation, Athens, Greece; ^2^Department of Biological Applications and Technology, University of Ioannina, Ioannina, Greece; ^3^Department of Pathology, Molecular Pathology Unit, Metaxa Cancer Hospital, Piraeus, Greece; ^4^Department of Nutrition and Dietetics, Harokopio University, Athens, Greece; ^5^Laboratory of General and Agricultural Microbiology, Agricultural University of Athens, Athens, Greece

**Keywords:** mushroom, genoprotection, anticancer, CpG methylation, immunomodulation, antioxidative mechanisms, DNA damage response, fecal microbiota

## Abstract

*Pleurotus eryngii* mushrooms are commercially cultivated and widely consumed due to their organoleptic properties, and the low caloric and high nutritional value. In addition, they contain various biologically active and health-promoting compounds; very recently, their genoprotective effect in Caco-2 cells after their fermentation by the human fecal microbiota was also documented. In the current study, the effect of *P. eryngii* pre- and post-fermentation supernatants in micronuclei formation was evaluated in human lymphocytes. In addition, the genoprotective properties of increasing concentrations of aqueous extracts from *P. eryngii* mushrooms (150, 300, 600 mg/kg) against the cyclophosphamide-induced DNA damage were studied in young and elderly female and male mice in bone marrow and whole blood cells. The ability of the highest dose (600 mg/kg) to regulate the main cellular signaling pathways was also evaluated in gut and liver tissues of female animals by quantifying the mRNA expression of *NrF2*, *Nfk*β, *DNMT1*, and *IL-22* genes. *P. eryngii* post-fermentation, but not pre-fermentation, supernatants were able to protect human lymphocytes from the mitomycin C-induced DNA damage in a dose-dependent manner. Similarly, genoprotection was also observed in bone marrow cells of mice treated by gavage with *P. eryngii extract.* The effect was observed in all the experimental groups of mice (young and elderly, male and female) and was more potent in young female mice. Overexpression of all genes examined was observed in both tissues, mainly among the elderly animals. In conclusion, *P. eryngii* mushrooms were shown to maintain genome integrity through protecting cells from genotoxic insults. These beneficial effects can be attributed to their antioxidant and immunomodulatory properties, as well as their ability to regulate the cell’s epigenetic mechanisms and maintain cell homeostasis.

## Introduction

The beneficial health properties of edible mushrooms have been known since ancient times. Mushrooms are a rich source of polysaccharides, mainly β-glucans, peptidoglucans, and proteoglucans (1, 2). They also contain a plethora of bioactive components, including proteins, chitins, unsaturated fatty acids, vitamins, triterpenic acids, lectins, statins, alkaloids, and polyphenolic compounds (1, 2). Mushrooms were found to be beneficial in preventing and treating chronic diseases, such as cardiovascular and neurodegenerative disorders, diabetes mellitus, hyperlipidemia, atherosclerosis, hypercholesterolemia, and cancer. Their biological activities are extensive, ranging from antioxidant, antimicrobial, prebiotic, immunomodulatory and anti-inflammatory to genoprotective, and anticarcinogenic (1–3). The latter properties, have been the focal point of intense research over the last decade.

Aqueous or organic extracts of mushrooms of various origins or their isolated ingredients were shown to have genoprotective properties against the insult of genotoxic and mutagenic carcinogens both *in vitro* (4–8) and *in vivo* (9). In a recent study, Chaga mushroom (*Inonotus obliquus*) polysaccharides exhibited genoprotective effects in UVB-exposed embryonic zebrafish (Danio rerio) through altered expression of DNA repair genes (10).

Mushroom fruit bodies, extracts or purified bioactive compounds have also been found to possess anticarcinogenic properties. Mushroom extracts exhibit *in vitro* anti-proliferating or pro-apoptotic activity against various cell lines (11–15), whereas mushrooms’ β-glucans, such as lentinan, schizophyllan, grifolan or polysaccharide-protein, and polysaccharide-peptide complexes, prevent oncogenesis by stimulating the immune system (16). A reduction in the density of lung metastases and lower expression of interleukin-6 (IL-6), cyclooxygenase-2 (COX-2), and nitric oxide synthase (NOS) were shown in mice with 4T1 triple-negative breast cancer (estrogen receptor; ER, progesterone receptor; PgR, human epidermal growth factor receptor-2; HER2) treated, orally, with a mixture of mushrooms (17). Furthermore, lentinan, a neutral polysaccharide purified from the extract of *Lentinula edodes* mycelia, is now approved to be used in clinical trials as cancer adjuvant therapy, and non-small cell lung carcinoma patients treated on chemo-immunotherapy of lentinan were shown to exhibit an increase in proliferation of cytotoxic T cells, accompanied by the rise in tumor necrosis factor-alpha (TNF-α), interferon gamma (IFN-γ), and interleukin-12 (IL-12) levels (18).

The genoprotective and anticarcinogenic properties of fungal bioactive compounds are frequently attributed to their action as radical scavengers (5, 6, 8) or as direct-acting immunomodulators (18). However, different bioactive compounds from mushrooms have been shown to alter the gut microbiome (GM), and recent research has demonstrated the fundamental role of the latter not only in colorectal carcinogenesis but also in almost every cancer (19, 20). Given that the host-microbiome crosstalk is very complex, changes in the gut microbial population caused by the diet or other confounders may promote or inhibit carcinogenesis (21), and microbiome excreted macromolecules, or small metabolites may act as tumor suppressors or promoters. However, studies on the genoprotective or anticarcinogenic properties of mushrooms fermented by the gut microbiota are scarce and focus only on the effects of fermentation of indigestible polysaccharides (22–26).

We recently investigated the genoprotective effects of edible mushrooms (*Pleurotus eryngii, Pleurotus ostreatus, and Cyclocybe cylindracea*) following their *in vitro* static batch culture fermentation by fecal inocula from elderly donors. We subsequently documented for the first time that fermented *Pleurotus eryngii* (and to a lesser extent *Pleurotus ostreatus)* mushrooms reduce the tert-butyl hydroperoxide (t-BOOH) induced oxidative DNA damage in Caco-2 human colon adenocarcinoma cells (27). In our study, elderly volunteers were recruited because their gradual age-associated decline of biological functions such as metabolism and immune responses make them more prone to developing various pathological conditions such as cancer (28–30). This finding provided substantial evidence that mushrooms may contain ingredients that, after fermentation by the human gut microbiota, protect genome integrity from genotoxic insults and may act as tumor suppressors. It is worth highlighting that precipitates and supernatants from the same *in vitro* reactions were previously examined for alterations in fecal microbiota composition (quantitative PCR) and in short-chain fatty acids (SCFA) concentrations (Gass Chromatography) respectively, during the 24-h fermentation processes. *P. eryngii* was shown to induce a strong lactogenic effect, whereas, all lyophilized mushrooms induced higher SCFA production than that induced by the same concentration (w/v) of the prebiotic polysaccharide inulin (3). Therefore, the beneficial effects of whole mushrooms or mushroom extracts need further investigation, specifically regarding their interactions with the gut microbiome and metabolome. In the current study, we extended the previously reported investigation on the genoprotective properties of *P. eryngii* fermentation supernatants (PE-FS) in human lymphocytes utilizing the Cytokinesis-block micronucleus (MN) assay. MN frequency in peripheral blood lymphocytes is predictive of the risk for various cancer types, making the Micronucleus test the most informative genotoxicity assay (31, 32). Furthermore, the genoprotective effect of an aqueous extract of *P. eryngii* mushroom (PE-E) was evaluated *in vivo*, using young (8–9 weeks old) as well as elderly (17–18 months old) mice of both genders. The extract’s ability to intervene and modulate main signaling pathways of the cell was also evaluated in gut and liver tissues of female animals by quantifying the mRNA expression of the hub genes nuclear factor erythroid 2–related factor 2 (*NrF2)*, Nuclear factor-kβ *(Nfk*β), DNA methyltransferase 1(*DNMT1)*, and Interleukin-22 (*IL-22)*.

## Materials and methods

### Chemicals and reagents

Ham’s F-10 was purchased from Biosera (Nuaille, France), mitomycin C and cytochalasin B were purchased from Cayman chemical company (Ann Arbor, MI, United States), Mushroom and Yeast Beta-Glucan assay kit (K-YBGL) was purchased from Megazyme (Bray, Ireland), Luna Universal PCR mix was purchased from New England Biolabs Inc. (Ipswich, MA, United States), Giemsa’s azur eosin methylene blue solution was purchased from Panreac Applichem (Castella del Valles, Spain), cyclophosphamide, DPX mounting medium, methanol, glacial acetic acid and formalin solution (neutral buffered, 10%) were purchased from Merck (Darmstadt, Germany), Fetal Bovine Serum (FBS), L-glutamine, M-MLV reverse transcriptase, Phytohemagglutinin M (PHA-M), and Trizol reagent were purchased from Thermo Fisher Scientific (Paisley, United Kingdom).

### Mushrooms: Cultivation, extract preparation, α- and β-glucan determination

Pure cultures of *Pleurotus eryngii* (PE) strain LGAM216 (Basidiomycota, Agaricales) are maintained in the Fungal Culture Collection of the Laboratory of General and Agricultural Microbiology (Agricultural University of Athens). Mushroom cultivation and batch *in vitro* fermentation were performed as previously described (3, 27). For the extraction, 50 g of lyophilized PE powder were suspended in 1 L H_2_O at 95°C for 24 h, under continuous stirring. The mixture was centrifuged at 4,500 rpm for 1 h; the supernatant was transferred to a new tube and evaporated using a rotary evaporator at 50°C. The concentrated supernatant was lyophilized to dryness, and the semisolid material obtained was kept at 4°C until use. α- and β-glucan content of *P. eryngii* extract (PE-E) was determined by K-YBGL kit (Megazyme) according to the manufacturer’s instructions. The extraction procedure resulted in an aqueous extract with total glucan content 43 ± 3.3% (w/w), α-glucan content 11 ± 0.7% (w/w), and β-glucan content 32 ± 2.7% (w/w). The lyophilized extract was dissolved in water at appropriate concentrations for the *in vivo* experiments.

### Subjects

#### Fecal sample donors

Five asymptomatic fecal donors (>60 years; three males, two females) were recruited, and the field study was conducted as previously described (3, 27). The study was performed according to the guidelines laid down in the Declaration of Helsinki and under the approval of the Bioethics Committee of Harokopio University, Athens, Greece (62-03/07/2018). Written informed consent was obtained from all fecal donors before their inclusion in the study.

#### Blood donors

Whole peripheral blood samples were obtained by venous puncture from four donors (two males, two females), for performing the *in vitro* micronucleus assay. The donors were asymptomatic, non-smoking volunteers of Greek nationality and ethnicity, 25–35 years old. Subjects had not been exposed to radiation or drugs 6 months before the study and were interviewed for the possible influence of confounding factors. The study was approved by the 6/30-05-2022 NHRF’s Research Ethics Committee.

### Batch *in vitro* fermentation

For the *in vitro* static batch culture fermentation, lyophilized *P. eryngii* powder was added to fresh fecal inocula obtained from all five volunteers, suspended in basal medium. Inoculum samples without the addition of mushroom powder from each volunteer were also included as negative controls (NC). Pre- and post-fermentation supernatants (FS) were obtained as described elsewhere (3, 27).

### Micronucleus assay *in vitro*

Five out of the eight matched pre- and post-fermentation supernatants (FS) obtained from the previous studies (3, 27) were examined for their modulatory effect on the genotoxicity of mitomycin C (reference genotoxic agent) in human lymphocytes using the Cytokinesis- block micronucleus assay. Whole blood from each of the four donors was treated independently with the PE-FS or NC-FS as previously described (33). Therefore, twenty independent experiments (five fecal donors × four blood donors) were performed for each of the conditions tested (pre-fermentation, post-fermentation). The procedure was as follows: 0.5 ml of peripheral blood was added to 8.3 ml Ham’s F-10 culture medium supplemented with 20% Fetal Bovine Serum, 1 mM L-glutamine and 4% v/v Phytohemagglutinin-M. The cultures were incubated at 37°C in a humidified incubator with 5% CO_2_ for 24 h. Pre- and post- fermentation supernatants were added at 1% v/v concentration in combination with the genotoxic agent mitomycin C (0.05 μg/ml). Cells treated with the medium or with only the genotoxic agent were also included as negative and positive controls, respectively. The cultures were incubated for 20 h, and then 6 μg/ml of the cytostatic agent cytochalasin B was added. The samples were collected 28 h later and centrifuged at 1,500 rpm for 10 min. Cell pellets were resuspended in 3 ml of hypotonic solution (Ham’s F-10: H_2_O, 3:1) and 5 ml of fixative solution (methanol: acetic acid, 5:1), under vigorous stirring. The samples were centrifuged at 1,500 rpm for 10 min and the cell pellets were resuspended in 5 ml of fixative solution. This procedure was repeated three times. Samples for microscopic observation were obtained by carefully dropping the cell suspension onto clean wet slides. Microscopic slides were stained with 10% Giemsa solution and covered with mounting media DPX and coverslips. The frequency of micronuclei in one thousand binucleated lymphocytes was evaluated under a light microscope for each sample.

### Micronucleus assay *in vivo*

#### Animals

CD1 mice purchased from Charles River were housed in the certified Animal Lab Facility of the National Hellenic Research Foundation (EL 25 BIO 031-033) under constant photoperiod cycle (12 h light, 12 h dark), humidity (55–60%), room temperature (22 ± 2°C) and free access to tap water and normal chow. Animal handling followed the regulations of the Federation of the European Laboratory Animal Science Association (FELASA) and the experimental design was approved by the Veterinary Office of Attica Prefecture (82837-30/01/2020) and the 7/15-06-2022 NHRF’s Research Ethics Committee.

#### Experimental design

This study was performed in accordance with the OECD guideline for the testing of chemicals #474 (1997): mammalian erythrocyte micronucleus test, as was updated in 2014 and published in 2016 (34). The genoprotective effects of *P. eryngii* hot water extract (PE-E) were assessed in young and elderly mice of both sexes. Seventy-two young mice aged 8–9 weeks (36 male and 36 female) and 72 elderly mice aged 17–18 months (36 male and 36 female) were separated by age and gender into groups of six animals. The animals were treated orally by gavage, once daily, with the mushroom’s hot water extract at doses of 150, 300, and 600 mg/kg for 14 days. Body weights were measured every 2 days, and the dose was adjusted to body weight changes. On the 14th day, the genotoxic agent cyclophosphamide (80 mg/kg) was also administered intraperitoneally for micronuclei formation. Twenty-four hours after CP administration, the animals were anesthetized for blood collection and killed by cervical dislocation for bone marrow isolation and tissue removal. Animals treated only with water (vehicle), cyclophosphamide, or PE-E for the same period were also included as controls.

#### Histopathological analysis

Liver and colon tissues were obtained only from elderly male and female mice of (a) the control group (C: 150 μl water administration, every day for 14 days), (b) the cyclophosphamide treated group (CP: 150 μl H_2_O for 14 days + 80 mg/kg cyclophosphamide at the 14th day) and (c) the group treated with the highest dose of PE-E and cyclophosphamide (CP + PE-E: 600 mg/kg mushroom extract for 14 days + 80 mg/kg cyclophosphamide at the 14th day). Tissues from each group were processed for histopathological examination. Liver and gut tissues were kept in 10% formalin solution before being embedded in paraplast, sectioned using a microtome (4 μm sections) and stained with Hematoxylin-Eosin (H/E). Three pathologists examined the tissue sections twice in a Nikon E400 microscope independently at different time points.

#### Micronucleus assay in the bone marrow and peripheral blood cells of mice

The Micronucleus assay in bone marrow was executed as previously described (35). Briefly, both femurs of the cervical dislocated animals were removed, and bone marrow cells were washed in fetal bovine serum. Bone marrow smears were prepared on clean glass slides, air-dried, fixed with absolute methanol, and stained with 10% Giemsa solution.

The Micronucleus assay in peripheral blood cells was performed according to the method described by Hayashi et al. (36). Briefly, peripheral blood was obtained from the superficial temporal vein, and blood smears were prepared instantly on clean glass slides, air-dried, fixed with absolute methanol, and stained with 10% Giemsa solution.

The micronucleus frequency in the bone marrow and whole blood cells was determined by counting the micronuclei in 1,000 polychromatic erythrocytes (PCEs) under a light microscope.

### RNA isolation, reverse transcription, and real-time qPCR

Total RNA was extracted using the Trizol reagent and reversed transcribed. The RNA quality was confirmed by agarose gel electrophoresis and the Agilent 4,150 Tapestation system. Real–time PCR was performed in 96-well plates using the Luna universal PCR mix in a CFX96 PCR thermal cycler (Biorad). The thermal cycling conditions comprised an initial denaturation step at 95°C for 60 s followed by 42 cycles at 95°C for 15 s and 60°C for 30 s. The comparative Ct method was used to calculate the relative gene expression (37). Expression levels of the genes of interest were normalized to the respective levels of GAPDH. Supplementary Table 1 shows the sets of real-time qPCR (RT-qPCR) primers used for each gene whose expression was studied.

### Statistical analysis

All statistical tests were performed by Sigmaplot 14 software. All data distributions passed the Shapiro–Wilk normality test. *In vitro* comparisons were analyzed using paired samples *t*-test. *In vivo* micronucleus assay data were analyzed by one-way ANOVA, *post hoc* Dunnett’s test for multiple comparisons. Gene expression comparisons were performed by student’s *t*-test with 95% confidence interval. *p ≤ 0.05 was considered as statistically significant.

## Results

### Genotoxic and genoprotective effects of pre- and post-fermentation supernatants *in vitro*

**FIGURE 1:**
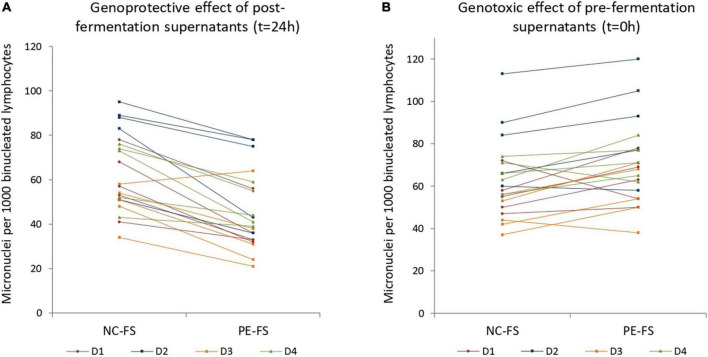
Genoprotective and genotoxic effects of **(A)** post- and **(B)** pre- fermentation supernatants in human lymphocytes. Human peripheral blood cells were treated with 1% v/v post- and pre- fermentation supernatants of NC and PE in combination with the mutagenic agent mitomycin C. Micronuclei formation was evaluated in 1,000 binucleated lymphocytes for each blood and fecal donor. **(A)** Genoprotective effect of 1% v/v post- fermentation supernatants of PE per blood and fecal donor (*t* = 24 h); **(B)** genotoxic effect of 1% v/v pre- fermentation supernatants of PE per blood and fecal donor (*t* = 0 h). NC-FS, fermentation supernatants without any additional carbon source; PE-FS, fermentation supernatants of *Pleurotus eryngii* mushroom; D1–D4, blood donors.

Peripheral blood mononuclear cells obtained from four blood donors (2 male, 2 female) were treated with 1% v/v fermentation supernatants in combination with the genotoxic agent mitomycin C (0.05 μg/ml). Five out of the eight matched pre- and post-fermentation supernatants (FS) obtained from previous studies (3, 27) were examined. Therefore, twenty independent experiments (five fecal donors × four blood donors) were performed for both PE treated (PE-FS) and untreated controls (NC-FS), and for each of the conditions tested (pre-fermentation, post-fermentation). Micronuclei formation was calculated in 1,000 binucleated lymphocytes per blood and fecal donor. Figure 1A represents the genoprotective effects of post-fermentation PE-FS. There was a consistent, highly significant (*t* = 7.5; *p* < 0.001) reduction of the micronuclei frequency formed (mean = −28.1%; SD = 15.4) for the PE-FS relative to the corresponding NC-FS. The genoprotective effect was fecal and blood donor independent as shown in Supplementary Figures 1A,B, ranging from −23.3 to −39.3% depending on the blood donor.

As previously observed in Caco-2 cells and batch *in vitro* fermentation of *P. eryngii* and other edible mushrooms (27), the pre-fermentation supernatants (PE-FS) exhibited a slight genotoxic effect (mean = 13.4%; SD = 17.0) when compared to the NC-FS (*p* = 0.003) (Figure 1B). However, the effect seems to be fecal and blood donor dependent as shown in Supplementary Figures 1A,B. These results indicate that *P. eryngii* and other mushrooms contain water-soluble constituents that are either genotoxic or become genotoxic through interactions with inoculum ingredients. These compounds are either degraded by the gut microorganisms or/and inactivated by their metabolic products that may exert genoprotective action.

### *In vivo* genoprotective properties of *Pleurotus eryngii* hot water extract

#### Body weight changes—Histopathology

**FIGURE 2:**
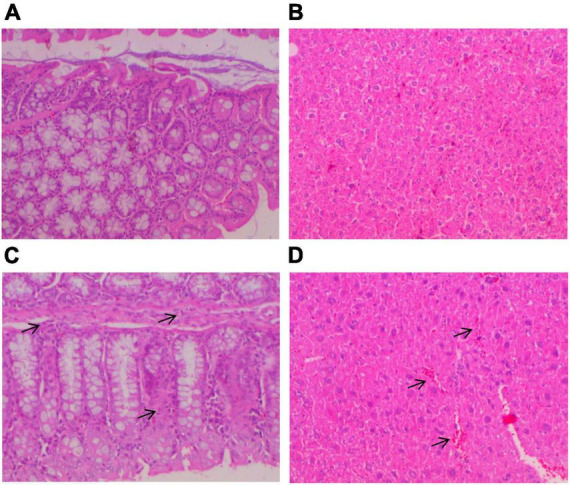
Histopathological analysis of colon and liver tissues of elderly mice. At the end of the treatment period, the mice were anesthetized and killed by cervical dislocation for tissue removal. Liver and gut tissues were kept in 10% formalin solution before being embedded in paraplast, sectioned using a microtome (4 μm sections) and stained with Hematoxylin-Eosin (H/E). The tissue sections were examined twice in a Nikon E400 microscope by three pathologists independently at different time points. **(A)** Colon and **(B)** liver tissues without any histopathological alterations; **(C)** colon tissue with small number of eosinophil leucocytes in the mucosal enteric crypts (arrows); **(D)** liver tissue with mild liver sinusoidal/canalicular dilatation (arrows).

There were no significant changes in body weight during the 14-day treatment period at any experimental group (data not shown). Histopathological examination of colon and liver tissues of the controls CP and CP + PE-E treated elderly male and female mice did not exhibit any significant tissue alterations (Figures 2A,B). A slight increase in the number of eosinophil leucocytes in the mucosal enteric crypts of the colon was sporadically observed in all groups (Figure 2C). Some of the CP + PE-E- treated animals also showed a mild liver sinusoidal/canalicular dilatation (Figure 2D).

#### Mn assay

The frequency of micronuclei formation in the bone marrow cells of young and elderly mice treated with different concentrations of *P. eryngii* hot water extract (PE-E) in combination with the alkylating agent cyclophosphamide is shown in Table 1. Administration of PE-E alone did not increase the number of micronuclei in polychromatic erythrocytes compared to the group which received water only. Young mice of both genders were more sensitive to the cyclophosphamide-induced genotoxicity and micronuclei formation than old mice of the same gender (*p* < 0.05). All groups treated with the highest concentration of the PE-E (600 mg/kg) showed a significant reduction in MN formation compared to the positive control group (CP) (*p* < 0.05) and exhibited a linear trend for micronuclei reduction as the dose increases which indicates that PE-E has genoprotective activities in mice of both gender and age groups. The genoprotective effect seems to be both age- and gender-dependent, being more potent among young and female mice, respectively. It should be noted that among the young females, statistically significant genoprotection was apparent even at the lower concentration of 150 mg/kg (Table 1).

**TABLE 1:** Number of micronucleated polychromatic erythrocytes (MNPCEs) per 1,000 polychromatic erythrocytes (PCEs) in bone marrow.

	Males				Females			
		
	MNPCE	MNPCE (% of CP)	*p*-values	*p* for linear trend	MNPCE	MNPCE (% of CP)	*p*-values	*p* for linear trend
**Young mice**								
Vehicle (H_2_O)	1 ± 1	2 ± 2			2 ± 2	3 ± 3		
PE-E 600 mg/kg	2 ± 1	4 ± 2			1 ± 1	2 ± 1		
CP 80 mg/kg	59 ± 3	100 ± 5			54 ± 9	100 ± 16		
CP + PE-E 150 mg/kg	51 ± 9	87 ± 15	0.252	<0.001	33 ± 6*	62 ± 12*	0.001	0.002
CP + PE-E 300 mg/kg	48 ± 9	82 ± 15	0.091		32 ± 12*	59 ± 22*	0.000	
CP + PE-E 600 mg/kg	37 ± 10*	62 ± 17*	< 0.001		36 ± 4*	67 ± 8*	0.004	
**Elderly mice**								
Vehicle (H_2_O)	2 ± 1	5 ± 4			3 ± 1	10 ± 5		
PE-E 600 mg/kg	2 ± 1	4 ± 3			2 ± 1	5 ± 3		
CP 80 mg/kg	40 ± 6	100 ± 16			32 ± 5	100 ± 15		
CP + PE-E 150 mg/kg	36 ± 5	91 ± 13	0.674	0.002	31 ± 4	97 ± 11	0.926	<0.001
CP + PE-E 300 mg/kg	35 ± 7	89 ± 18	0.526		28 ± 3	87 ± 11	0.127	
CP + PE-E 600 mg/kg	26 ± 7*	65 ± 18*	0.004		21 ± 1*	66 ± 4*	0.000	

MNPCEs, micronucleated polychromatic erythrocytes; PCEs, polychromatic erythrocytes; CP, cyclophosphamide; PE-E, Pleurotus eryngii hot water extract. Values are expressed as the mean ± SD of six mice in each group. *p < 0.05 (one-way Anova, Dunnett test).

The frequency of micronuclei formation in whole blood cells of young mice treated with different concentrations of PE-E was also examined. The differences observed in micronuclei formation were not significant (Supplementary Table 2), mainly due to the high deviations observed between animals of the same group. For that reason, the micronuclei assay in whole blood cells of the older animals was not performed.

#### Antioxidant and immunoregulatory effects of *Pleurotus eryngii* mushroom extract administration in mice

**FIGURE 3:**
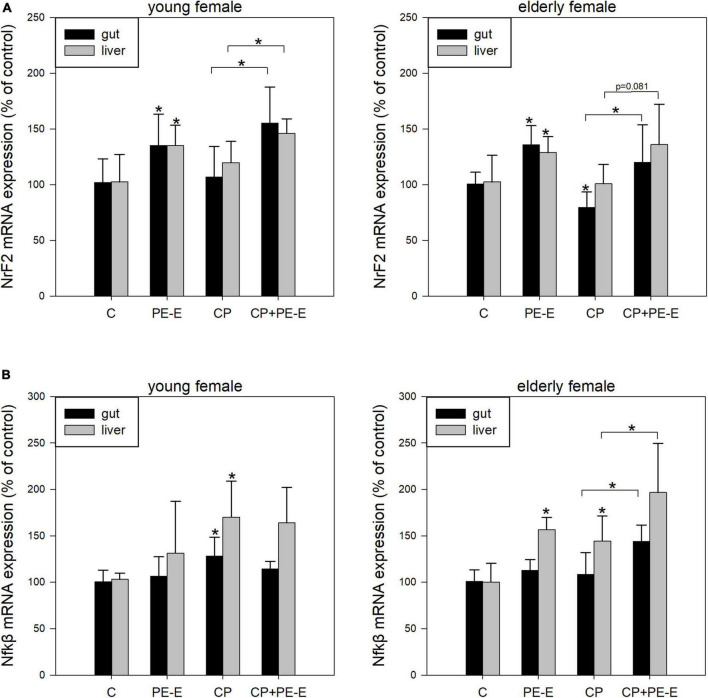
mRNA expression levels of **(A)** *NrF2* and **(B)** *Nfk*β in gut and liver tissues of young and elderly female mice. C, negative control group treated with water for 14 days; PE-E, *Pleurotus eryngii* control group treated with 600 mg/kg *P. eryngii* mushroom extract for 14 days; CP, positive control group treated with water for 14 days and 80 mg/kg cyclophosphamide the 14th day; CP+PE-E, administration of 600 mg/kg *P. eryngii* mushroom extract for 14 days and 80 mg/kg cyclophosphamide the 14th day. All values are expressed as the mean ± SD of six mice in each group. *p < 0.05 compared to control (water) (*t*-test).

In order to investigate the possible involvement of antioxidant or immunoregulatory mechanisms in the genoprotective ability of *P. eryngii* mushroom extract (PE-E), the mRNA expression levels of the hub genes *NrF2* and *Nfk*β for the respective processes were quantified in gut and liver tissues. The RNA quantitation analysis was performed only for the female mice, which showed more responsiveness to the PE-E in the micronuclei assay. The groups selected were the control C (H_2_O), PE-E (600 mg/kg), CP and CP + PE-E (600 mg/kg). Figures 3A,B represent the expression levels of *NrF2* and *Nfk*β in young and elderly female mice. The effect of PE-E in the expression of *NrF2* appears to be both tissue and age-independent (Figure 3A). Administration of the PE-E alone or in combination with CP significantly increased the levels of *NrF2* mRNA in both tissues relative to those of the control groups C and CP, respectively. The expression of *NrF2* gene was reduced in the gut of CP treated elderly mice (*p* < 0.05), which however was restored to approximately the control levels in mice of the CP + PE-E group. The effect of PE-E in the expression of *Nfk*β appeared to be age-dependent and was more pronounced among the elderly mice (Figure 3B). In young mice, administration of the PE-E alone or in combination with CP did not alter the levels of *Nfk*β relative to the control groups, C and CP, respectively. Among the young mice, only the CP-treated group showed in both tissues elevated expression of *Nfk*β relative to all the other groups. In elderly female mice, administration of PE-E alone upregulated *Nfk*β in the liver, whereas its administration in combination with CP significantly increased the levels of *Nfk*β mRNA in both tissues relative to those of the respective control CP-treated groups (Figure 3B).

#### Epigenetic remodeling and *Pleurotus eryngii* mushroom extract administration in mice

**FIGURE 4:**
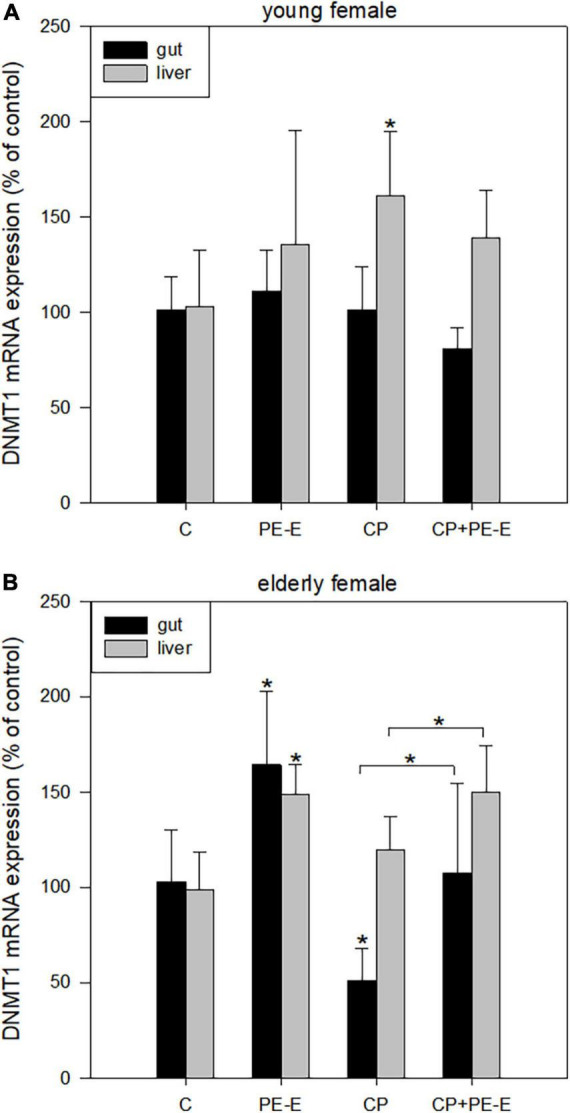
mRNA expression levels of *DNMT1* in gut and liver tissues of **(A)** young and **(B)** elderly female mice. C, negative control group treated with water for 14 days; PE-E, *Pleurotus eryngii* control group treated with 600 mg/kg *P. eryngii* mushroom extract for 14 days; CP, positive control group treated with water for 14 days and 80 mg/kg cyclophosphamide the 14th day; CP+PE-E, administration of 600 mg/kg *P. eryngii* mushroom extract for 14 days and 80 mg/kg cyclophosphamide the 14th day. All values are expressed as the mean ± SD of six mice in each group. *p < 0.05 compared to control (water) (*t*-test).

The ability of the extract to regulate epigenetic mechanisms, such as DNA methylation, was also evaluated by quantification of the mRNA expression levels of the *DNMT1* gene, which is responsible for the maintenance and possibly the *de novo* CpG methylation of transposable elements (38). An age-dependent effect of PE-E treatment was also observed for *DNMT1* expression levels with the young animals showing no differences in *DNMT1* expression between PE-E treated and relative control groups in both tissues (Figure 4A). Among the young mice only the CP treated group showed elevated expression of *DNMT1* in the liver relative to all the other groups. On the contrary, treatments with PE-E alone or in combination with CP caused a significant induction in *DNMT1* expression levels in gut and liver tissues of elderly female mice compared to the respective controls (Figure 4B). In addition, as also shown in Figure 4B, PE-E seems to have the ability in the CP + PE-E group of animals to restore the reduced expression levels of *DNMT1* in the gut of elderly females treated only with CP.

#### DNA damage response of *Pleurotus eryngii* mushroom extract administration in mice

**FIGURE 5:**
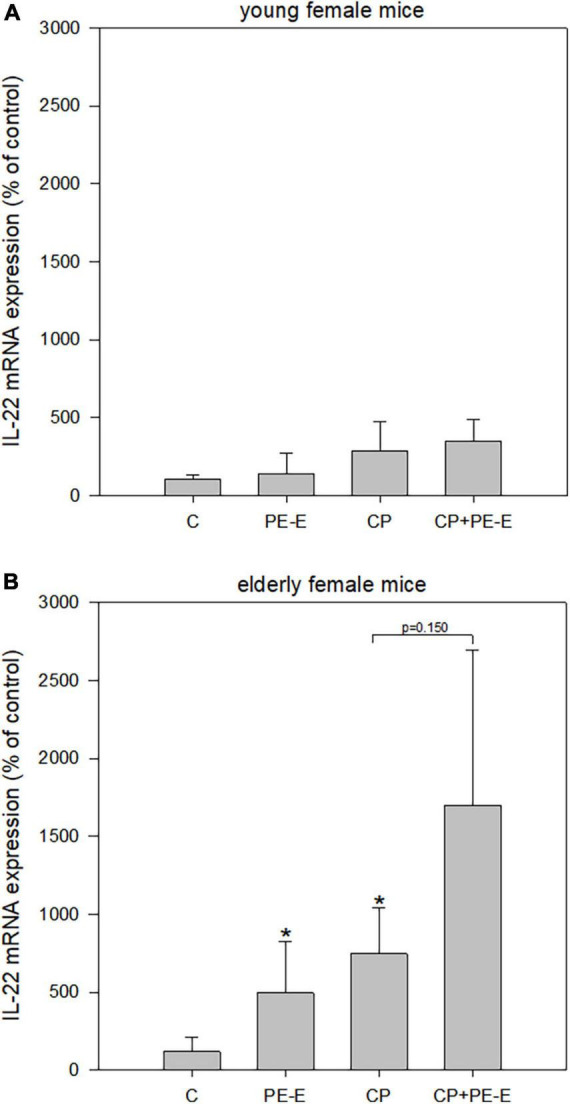
mRNA expression levels of *IL-22* in gut tissue of **(A)** young and **(B)** elderly female mice. C, negative control group treated with water for 14 days; PE-E, *Pleurotus eryngii* control group treated with 600 mg/kg *P. eryngii* mushroom extract for 14 days; CP, positive control group treated with water for 14 days and 80 mg/kg cyclophosphamide the 14th day; CP+PE-E, administration of 600 mg/kg *P. eryngii* mushroom extract for 14 days and 80 mg/kg cyclophosphamide the 14th day. All values are expressed as the mean ± SD of six mice in each group. *p < 0.05 compared to control (water) (*t*-test).

*Pleurotus eryngii* mushrooms potential in maintaining cell homeostasis was evaluated by the quantification of *IL-22* mRNA expression levels in gut tissues only (Figure 5), since its expression in the liver was not detectable (data not shown). Once more the age effect was prominent with the young animals showing only α minor non-significant increase (*p* > 0.05) in *IL-22* expression in PE-E treated animals relative to the respective controls. On the contrary, treatments with PE-E alone or in combination with CP caused overexpression of *IL-22* compared to the control groups in elderly females. The induction in the expression levels of *IL-22* in the CP + PE-E relative to the CP group was however not significant (*p* = 0.150) due to the high interindividual variations in the expression levels among the animals of the same group.

## Discussion

Edible mushrooms contain a variety of bioactive compounds, such as polysaccharides, sterols, coumarins, and polyphenols, with numerous biological activities (22, 39, 40). The genoprotective and anticarcinogenic properties of fungal bioactive compounds are frequently attributed to their direct action as radical scavengers or immunomodulators (5, 6, 18). However, over the last years, an indirect mode of action that implicates the gut microbiome as a mediator is gaining considerable ground (19, 20). Although not adequately addressed so far, the mushrooms’ anticancer properties may be due to their fermentation by the human gut microbiota and the metabolites produced in the process (41, 42).

### Batch *in vitro* fermentation and comparative genoprotection study of pre- and post-fermentation supernatants of *Pleurotus eryngii*

*Pleurotus eryngii* mushroom powder was fermented *in vitro* by fecal inocula of healthy elderly volunteers for 24 h. Pre- and post-fermentation supernatants were collected, and their effect on DNA damage induced by the genotoxic agent mitomycin C was assessed by Cytokinesis block micronucleus assay in human lymphocytes. According to our results, even at the low concentrations used (1% v/v), post-fermentation supernatants decrease remarkably (28.1%) the micronuclei frequency in human lymphocytes. The reduction of micronuclei was consistent throughout all the experiments and fecal and blood donor-independent (Supplementary Figure 1). On the contrary, the pre-fermentation supernatants of PE (PE-FS) exhibited a moderate genotoxic effect (13.4%) compared to the NC pre-fermentation supernatants (NC-FS); the effect was fecal and blood donor dependent (Supplementary Figure 2). We have previously demonstrated using the comet assay that post-fermentation supernatants of *P. eryngii* mushroom exhibit genoprotective effects against the genotoxic agent tert-butyl-hydroperoxide (t-BOOH) in human small intestinal cancer cells (Caco-2) (27). In the same study, we showed that pre-fermentation supernatants of another mushroom, *Cyclocybe cylindracea*, were significantly genotoxic in Caco-2 cells. At the same time, the respective *P. eryngii* fermentation supernatant was also moderately but not significantly genotoxic. Therefore, *P. eryngii* mushrooms may contain water-soluble ingredients that are either genotoxic *in vitro* or ameliorate the well-known genotoxic activity of the aqueous phase of human stool (43).

Little is known about the protective role of mushrooms on DNA damage, after their fermentation in the gut (22–26), and the main question arising is which metabolites are being associated with the genoprotective effect. During the fermentation process of *Pleurotus eryngii* mushrooms or their extracts by gut microbiota, short chain fatty acids (SCFAs) such as acetate, propionate and butyrate are produced (44). SCFAs are involved in many biological processes and might be associated with genoprotective properties (23), such as those we observed for fermented *P. eryngii.* The matched pre- and post-fermentation supernatants (FS) examined in the current study and their respective precipitates were previously examined for alterations in short-chain fatty acids (SCFA) concentration and in fecal microbiota composition respectively, during the 24-h fermentation processes (3, 27). *P. eryngii* was shown to induce higher SCFA production than similar concentrations (w/v) of the prebiotic polysaccharide inulin (3) possibly because it exhibits a strong lactogenic effect. Furthermore, our preliminary untargeted NMR-based metabolomics analysis of PE-FS showed a remarkable increase of short-chain fatty acids (SCFAs), namely acetate, propionate, butyrate, and formate, and enhanced production of trimethylamine (TMA) as well as of the neurotransmitter gamma-aminobutyric acid (GABA) (27).

### *In vivo* genoprotective effect of *Pleurotus eryngii*

T he genoprotective effect of *P. eryngii* hot water extract (PE-E) was investigated in young (8–9 weeks old) and elderly mice of both sexes in bone marrow cells. The old mice were 17-18 months old, corresponding to 56–60 human years. DNA damage was induced by cyclophosphamide (CP), an alkylating agent, which is metabolized in the liver by cytochromes P450. CP is an established genotoxic agent since it can cause DNA damage when metabolized, leading to chromosome breaks, micronuclei (MN) formation, and cell death (45, 46). In the present work, cyclophosphamide was administered intraperitoneally at a dose of 80 mg/kg. This dosing regimen induces micronuclei formation without causing any tissue damage, as confirmed by histopathological examination of liver and gut tissues. All animal groups treated with PE-E only (highest dose; 600 mg/kg), exhibited micronuclei frequency similar to the controls, indicating that *P. eryngii* mushroom extract is not genotoxic *in vivo*. In addition, histopathological examination of randomly selected animals (groups of old mice) did not show significant gut or liver alterations. The small increase in the number of eosinophil leucocytes in the mucosal enteric crypts of the colon and the mild liver sinusoidal/canalicular dilatation observed in some animals could be attributed to their old age. The number of micronuclei after CP administration was lower in aged mice compared to young and in aged females compared to aged male mice. Variations on DNA damage between genders have been previously reported by the Collaborative study group for the micronucleus test (47). According to this study, male CD-1 mice treated with cyclophosphamide were more prone to micronuclei formation compared to females. All groups treated with the highest concentration of the extract (CP + PE-E 600 mg/kg) showed a significant reduction in MN formation compared to the positive control group (CP) (*p* < 0.05) (Table 1). Furthermore, administration of the *P. eryngii extract* at doses of 150, 300, and 600 mg/kg/day for 14 days prevented the formation of MN in the bone marrow cells of CP-treated mice of all groups in a dose-dependent manner as the p values for linear trend indicated. Therefore, when administered by gavage in mice, PE-E exerts genoprotective activities in both male and female, young and elderly animals.

Our results are in agreement with two recent studies, where extracts of the mushrooms *Phellinus baumii* and *Pleurotus ostreatus* (ethanolic and aqueous extracts, respectively) prevented the formation of MN in the bone marrow cells of CP-treated mice in a dose-dependent manner (39, 48).

Moreover, significant age- and gender-dependent differences in the genoprotection were observed between the groups. PE-E exhibited higher genoprotection in the female and young mice than in male and old, respectively. It has been reported that the response to genotoxic agents is strain-, sex- and tissue-dependent, and the differences observed between males and females are attributed to the differential expression of genes involved in DNA repair mechanisms, such as *Mpg*, *Xrcc1*, and *Mgmt* (49). A similar gender-dependent response to genoprotective agents may exist. Another possible explanation for these differences may be the influence of sex hormones on cytochrome P450 (50).

It is generally known that aging affects various biological functions of the organism, enhancing the accumulation of DNA adducts and the formation of micronuclei. Contrary to the above, our experiments showed a reduction in micronuclei formation in the bone marrow cells of CP-treated elderly mice compared to younger mice. These differences may be due to the liver’s reduced xenobiotic (including CP) metabolizing capacity upon aging (51). We also found that the mushroom extract does not protect older mice from CP induced- DNA damage at the same level as younger mice. This finding was also observed in (1→3) (1→6)-β-D-glucan botryosphaeran treated mice (9) and could be attributed to the decreased gut microbiota diversity and increased immunosenescence that comes with aging (52).

The micronucleus assay is widely used in bone marrow cells and, to a lesser extent in peripheral blood cells (53, 54). Therefore, we also examined the frequency of micronuclei formation in whole blood cells among young mice. Due to the high deviations observed in the micronuclei frequency among the animals of the same group and the non-significant changes (Supplementary Table 2), the experiment was not repeated for the elderly mice groups. It is generally known that polychromatic erythrocytes (PCEs) need more time to appear in the circulating blood compared to the PCEs in the bone marrow. Since animals were killed 24 h after CP administration, an appropriate time point for the evaluation of the CP’s acute effect in bone marrow (55, 56), the frequency of micronucleated polychromatic erythrocytes was low and not sufficient to produce reliable results.

### Regulation of main signaling pathways by *Pleurotus eryngii* extract

To investigate the mechanisms involved in the genoprotective ability of *P. eryngii* extract (PE-E), the mRNA expression levels of the genes *NrF2* and *Nfk*β, *DNMT1* and *IL-22* were quantified in gut and liver tissues in the female mice, which were shown to be more responsive to the genoprotective activity of PE-E. NrF2 (nuclear factor erythroid 2-related factor 2) is a major transcriptional factor involved in antioxidant response pathways. Upon activation by oxidative or genotoxic stressors, NrF2 accumulates into the nucleus and binds to antioxidant response elements (ARE), thus regulating the expression of antioxidant genes (57). Nfkβ is an essential transcriptional factor in main cellular responses, such as immunity, inflammation, homeostasis, and cell death. During activation by various factors, such as DNA damage agents, Nfkβ translocates to the nucleus, binds to specific gene promoters, and regulates the expression of hundreds of genes (58). *DNMT1* is one of the hub genes that orchestrate one of the most important epigenetic modifications, CpG DNA methylation, which plays key roles in regulating gene expression, genomic imprinting, X chromosome inactivation, and tumorigenesis. *DNMT1* gene is responsible for the maintenance of CpG methylation across the genome and possibly the *de novo* CpG methylation of transposable elements (38). IL-22 is a member of the IL-10 family of cytokines. It is produced mainly by T cell subsets and type 3 innate lymphoid cells and is an important regulator of the immune response, protection of intestinal barrier, and maintenance of gut homeostasis and microbial composition (59).

The effect of administration of PE-E alone or combined with CP on the expression of the genes mentioned above, *NrF2* exempted, was age-dependent. The effect was significant only among the elderly mice. This was not surprising since, upon external stimuli, an age-dependent differential gene expression response is commonly observed in both animal and human studies (60–62). Furthermore, differential age-related mRNAs expression was also observed despite the similar micronuclei frequency observed in both age groups treated with CP and 600 mg/kg PE-E. Similarly, Thalacker-Mercer et al. (63) reported differential genomic responses in old vs. young humans despite similar levels of modest muscle damage after resistance loading.

Pre-treatment with PE-E alone or combined with CP caused a remarkable induction in *NrF2* expression levels in both tissues of young and elderly female mice compared to the respective control groups (water and CP) (Figure 3A). In addition, *NrF2* expression after CP administration was significantly reduced in the gut of elderly animals. However, it was restored to the control levels in the animals in which CP was co-administered with PE-E. Although our results are restricted only at the gene expression level, they support previous studies, which showed that mushroom extracts possess strong antioxidant properties through their ability to regulate the NrF2 signaling pathway and to ameliorate the oxidative effects caused by CP administration by regulating the transcription of glutathione peroxidase (GSH-Px), nicotinamide adenine dinucleotide phosphate [NAD(P)H] quinine oxidoreductase-1 (NQO-1), heme oxygenase-1 (HO-1), superoxide dismutase 2 (SOD2), and catalase (CAT) antioxidant enzymes (64, 65).

In elderly mice, administration of the PE-E alone upregulated *Nfk*β expression in the liver, whereas its administration in combination with CP significantly increased the levels of *Nfk*β in both tissues relative to those of the CP treated control groups (Figure 3B). According to previous studies, various hot water extracts of mushrooms possess immunomodulatory properties through the stimulation of immune cells, such as macrophages (66–70), dendritic (71) or B cells (72) *via* activation of toll-like receptor 4 (TLR4), mitogen activated protein kinase (MAPK) and Nfkβ signaling pathways. Our results indicate that *P. eryngii* could probably modulate the expression of genes involved in immune regulation through the activation of Nfkβ signaling pathway, which is in agreement with very recent findings by our group. We reported that the *in vitro* fermentation of *P. eryngii* mushrooms by fecal microbiota of healthy elderly volunteers clearly affected immune response, as indicated by the altered gene expression and secretion levels of pro- (TNF-α, IL-1β) and anti-inflammatory cytokines (IL-10, IL-1Rα) in human macrophages while fermentation supernatants induced immunophenotypic changes in T helper 2 (Th2) and myeloid lineage cells in their peripheral blood mononuclear cells (PBMCs) (73).

Treatments with PE-E alone or in combination with CP caused a significant induction in *DNMT1* expression levels in gut and liver tissues of elderly female mice compared to the respective controls (C and CP) (Figure 4B). It has recently been shown that alterations in gut microbiota and its metabolites’ composition may affect the epigenetic mechanisms of gene expression. Gut microbiota has recently been identified as a critical mediator of the intestinal inflammatory response through epigenetic reprograming (74). It was also shown that SCFAs produced by gut microbiota regulate endothelial function and reduce inflammation by inhibiting the histone deacetylases (HDACs), a key epigenetic enzyme (75, 76). The current study, to the best of our knowledge, is the first study showing that ingestion of a mushroom extract causes alterations in *DNMT1* expression in mice. Furthermore, it is worth noting that in our study PE-E, when co-administered with CP, seem to have the ability to restore the reduced expression levels of *DNMT1* in the gut of CP-treated elderly animals. The only partially relevant work is that of Sharma and Tollefsbol (77) where a mixture of two dietary phytochemicals (Sulforaphane and Genistein) and butyrate inhibits *DNMT1* expression in MDA-MB-231 and MCF-7 breast cancer cells.

In the present work, treatments of elderly animals with PE-E alone or in combination with CP caused overexpression of *IL-22* in the gut compared to the respective control groups (Figure 5). Recent studies have revealed that upon exposure to genotoxic agents, IL-22 promotes the activation of Ataxia Telangiectasia Mutated pathway (ATM) and the initiation of DNA damage response (DDR) in colon stem cells. In addition, it has been shown that innate lymphocytes regulate the expression of *IL-22* and, consequently, the activation of DDR through the AhR expression in response to genotoxic dietary factors (78). Butyrate promotes IL-22 production, enhancing butyrate’s protective effect in intestinal inflammation in mice (79). Considering the aforementioned, the induction of *IL-22* expression in our experiments probably promotes the activation of the DDR, which is highly upregulated in the CP + PE-E groups, resulting in the inhibition of the DNA damage caused by CP and the reduction in MN formation. These effects could be associated with the production of SCFAs, since their production affects T cell populations (80), and consequently, *IL-22* levels.

## Conclusion

The current study focused on exploring the genotoxic/genoprotective properties of the mushroom *P. eryngii.* Our results indicate that *P. eryngii*, fermented by fecal inocula *in vitro* or ingested *in vivo*, exerts genoprotective properties against the genotoxic insult of well-known DNA damaging agents. In order to elucidate the role of the microbiome and its metabolome in the observed genoprotection of fermented PE, extensive 16S Next Generation Sequencing (NGS) metagenomic analysis and full-scale NMR-based metabolomics analysis in both the *in vitro* and *in vivo* experiments are currently underway. The quantification of mRNA expression of the hub genes *NrF2, Nfk*β, *DNMT1*, and *IL-22* in mice gut and liver tissues may suggest possible mechanisms for the observed genoprotection *via* modulation of the antioxidant and immunosurveillance responses of the cells, alterations in epigenetic mechanisms, maintenance of cell homeostasis, and promotion of the DNA damage response pathways.

## Data availability statement

The original contributions presented in this study are included in the article/Supplementary material, further inquiries can be directed to the corresponding author.

## Ethics statement

The studies involving human participants were reviewed and approved by Bioethics Committee of Harokopio University, Athens, Greece (62-03/07/2018) and by NHRF’s Research Ethics Committee. The patients/participants provided their written informed consent to participate in this study. The animal study was reviewed and approved by the Veterinary Office of Attica Prefecture (82837-30/01/2020) and the 7/15-06-2022 NHRF’s Research Ethics Committee.

## Author contributions

AB contributed to the conceptualization, data curation, methodology, validation, formal analysis, and writing the original draft. PM, G-MS, ES, CV, GS, EM, GK, GZ, and AK contributed to the formal analysis, data curation, and methodology. VP contributed to the conceptualization, funding acquisition, project administration, and reviewing and editing the manuscript. PG contributed to the conceptualization, methodology, supervision, investigation, validation, and writing, reviewing, and editing the manuscript. All authors contributed to the article and approved the submitted version.
