# Supplementary material for: Genoprotective activity of the Pleurotus eryngii mushrooms following their in vitro and in vivo fermentation by fecal microbiota
Source: Front Nutr. 2022 Aug 23;9:988517. doi: 10.3389/fnut.2022.988517 (PMC9445615; doi:10.3389/fnut.2022.988517)
Supplement: Supplementary file 1 [file Data_Sheet_1.pdf]

Supplementary Tables and Figures

Supplementary Table 1. Primer pairs for RT-qPCR

Mouse *Nfkβ*:	Forward 5’-CTGAACAAAATGCCCCACGG- 3’<br>Reverse 5'- TTCCTCCTTTGGGACGATGC- 3’
Mouse *NrF2*:	Forward 5’-AGCCAGCTGACCTCCTTAGA- 3’<br>Reverse 5'-AGTGACTGACTGATGGCAGC- 3’
Mouse *DNMT1*:	Forward 5’-ATGGCGTCATAGCCCATAAG- 3’<br>Reverse 5' -TGCACAGGAACAGACTCCAC- 3’
Mouse *IL-22*:	Forward 5’ -GCTCATCGGGGAGAAACTGT- 3’<br>Reverse 5'-TGTAGGGCTGGAACCTGTCT- 3’
Mouse *GAPDH*:	Forward 5’-CATGGCCTTCCGTGTTCCTA- 3’<br>Reverse 5'-CCTGCTTCACCACCTTCTTGAT- 3’

Supplementary Table 2. Number of micronucleated polychromatic erythrocytes (MNPCEs) per 1000 polychromatic erythrocytes (PCEs) in whole blood cells.

Young mice	Males				Females			
	MNPCE	MNPCE (% of CP)	p values	p for Linear trend	MNPCE	MNPCE (% of CP)	p values	p for Linear trend
Vehicle (H_2O)	11±2	26±5			7±4	23±14		
PE 600 mg/kg	4±2	10±4			6±3	19±9		
CP 80 mg/kg	41±6	100±14			29±10	100±33		
CP + PE-E 150 mg/kg	39±8	96±20	0.990		21±8	72±27	0.252	
CP + PE-E 300 mg/kg	25±10	61±23	0.024	0.058	27±9	94±31	0.970	0.199
CP + PE-E 600 mg/kg	34±13	82±32	0.479		33±6	115±20	0.659	

MNPCEs: micronucleated polychromatic erythrocytes; PCEs: polychromatic erythrocytes; CP: cyclophosphamide; PE-E: *Pleurotus eryngii* extract. Values are expressed as the mean ± SD of six mice in each group. * $p < 0.05$ (one-way Anova, Dunnett test).

(A)

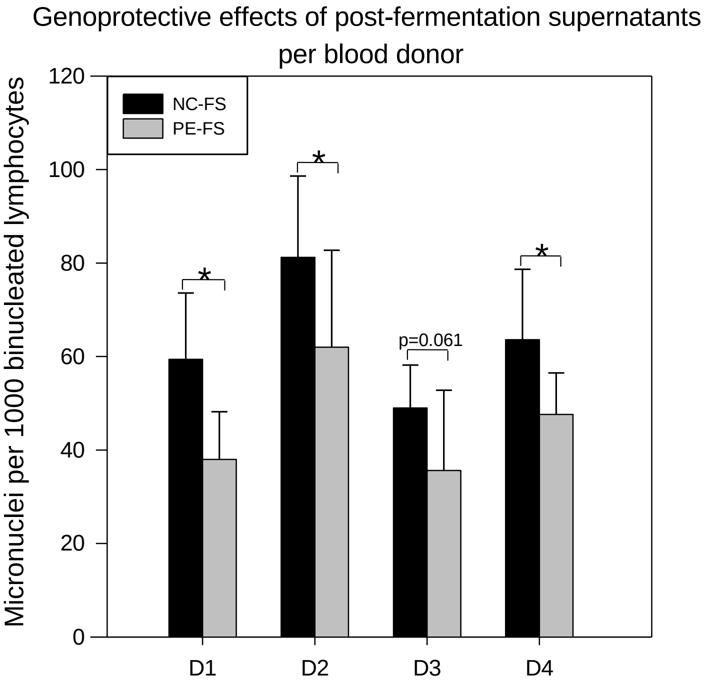

(B)

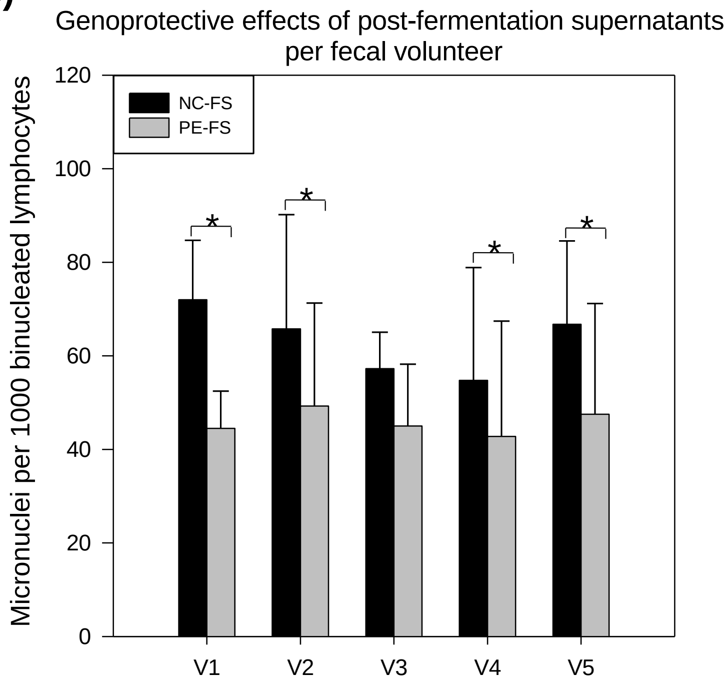

Supplementary Figure S1: Genoprotective effects of post-fermentation supernatants in human lymphocytes. Human peripheral blood cells were treated with 1% v/v post- fermentation supernatants of NC-FS and PE-FS in combination with the mutagenic agent mitomycin C. Micronuclei formation was evaluated in 1000 binucleated lymphocytes for each blood and fecal donor. Genoprotective effect of 1% v/v post- fermentation supernatants of PE-FS (A) per blood donor and (B) per fecal volunteer. NC-FS: Fermentation supernatants without any additional carbon source; PE-FS: fermentation supernatants of *Pleurotus eryngii* mushroom; D1-D4: blood donors; V1-V5: Fecal volunteers. All values are expressed as the mean ± SD. * $p < 0.05$ compared to NC-FS (paired t-test, Anova).

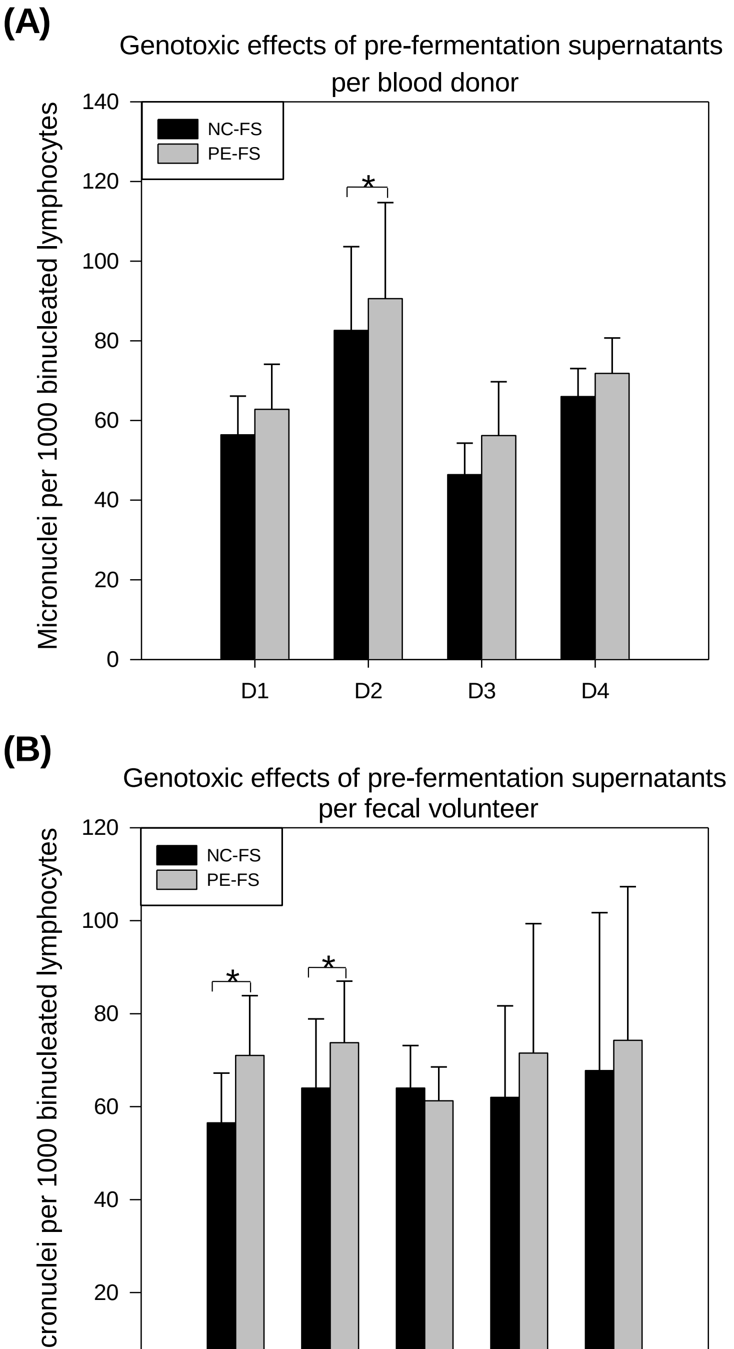

Supplementary Figure S2. Genotoxic effects of pre-fermentation supernatants in human lymphocytes. Human peripheral blood cells were treated with 1% v/v pre-fermentation supernatants of NC-FS and PE-FS in combination with the mutagenic agent mitomycin C. Micronuclei formation was evaluated in 1000 binucleated lymphocytes for each blood and fecal donor. Genotoxic effect of 1% v/v pre-fermentation supernatants of PE-FS (A) per blood donor and (B) per fecal donor. NC-FS: Fermentation supernatants without any additional carbon source; PE-FS: fermentation supernatants of *Pleurotus eryngii* mushroom; D1-D4: blood donors; V1-V5: Fecal volunteers. All values are expressed as the mean ± SD. * $p < 0.05$ compared to NC-FS (paired t-test, Anova).
